# *Zwartia vadi* sp. nov., a Novel Species of the GKS98 Cluster Isolated from a Stream, and the Reclassification of ‘Achromobacter Panacis’ as *Zwartia panacis* comb. nov

**DOI:** 10.3390/microorganisms11092150

**Published:** 2023-08-24

**Authors:** Miri S. Park, Ilnam Kang, Jang-Cheon Cho

**Affiliations:** 1 Department of Biological Sciences and Bioengineering, Inha University, Incheon 22212, Republic of Korea; jea9911@inha.ac.kr; 2 Center for Molecular and Cell Biology, Inha University, Incheon 22212, Republic of Korea

**Keywords:** *Zwartia vadi* sp. nov., *Zwartia panacis* comb. nov., polyphasic taxonomy, whole-genome sequencing, GKS98 cluster

## Abstract

A Gram-stain-negative, aerobic, motile by gliding, and rod-shaped bacterium, designated IMCC34845^T^, was isolated from a freshwater stream in the Republic of Korea. The results of 16S rRNA gene-based phylogenetic analyses showed that strain IMCC34845^T^ was affiliated with the genus *Zwartia* and was most closely related to ‘Achromobacter panacis’ DCY105^T^ (100%) and *Zwartia hollandica* LF4-65^T^ (98.9%). The whole-genome sequence of strain IMCC34845^T^ was 3.2 Mbp in size with a 51.5% DNA G+C content. The average nucleotide identity (ANI) and digital DNA-DNA hybridization (dDDH) values between strain IMCC34845^T^ and ‘Achromobacter panacis’ KCTC 42751^T^ were 77.7% and 20.7%, respectively, revealing that they are independent species. Moreover, the strains IMCC34845^T^ and KCTC 42751^T^ exhibited ≤ 72.5% ANI and ≤18.5% dDDH values with closely related species *Zwartia hollandica* LF4-65^T^, further supporting that the two strains represent each novel species of the genus. The major respiratory quinone of strain IMCC34845^T^ was ubiquinone-8 (Q-8), and the predominant cellular fatty acids were C_16:0_ (41.3%) and C_17:0_ cyclo (34.5%). The major polar lipids of the strain were diphosphatidylglycerol, phosphatidylglycerol, phosphatidylethanolamine, unidentified phospholipids, and unidentified aminolipids. Based on the phylogenetic, genomic, physiological, and chemotaxonomic characteristics, strain IMCC34845^T^ was considered to represent a novel species within the genus *Zwartia*, for which the name *Zwartia vadi* sp. nov. is proposed. The type of strain is IMCC34845^T^ (=KCTC 92920^T^ = NBRC 114902^T^). Furthermore, based on the taxonomic data, ‘Achromobacter panacis’ is proposed to be reclassified as *Zwartia panacis* comb. nov.

## 1. Introduction

The GKS98 [1] or betIII [2] cluster, belonging to the bacterial family *Alcaligenaceae* [3], has been recognized as one of the typical bacterial assemblages in freshwater ecosystems [2]. The GKS98 cluster was originally designated based on 16S rRNA sequences retrieved from Lake Gossenköllesee [4]. Subsequently, its presence has been identified in diverse freshwater systems through numerous cultivation-independent investigations [1,2,5,6,7,8]. The first proposal of the taxa affiliated with the GKS98 cluster was suggested by Hahn et al. in 2022 [9], revealing that the cluster represents three genera: *Sheuella*, *Jezberella*, and *Zwartia*. The classification was based on the proposal of two novel species, *Jezberella montanilacus* and *Zwartia hollandica*, and the reclassification of *Orrella amnicola* [10] as *Sheuella amnicola*. Although some other strains of the cluster have previously been isolated [11,12], there are currently only the three validly described species within the cluster.

As of July 2023, the genus *Zwartia* is represented by a sole species, *Zwartia hollandica*, exhibiting characteristics of an aerobic, unpigmented, rod-shaped, and chemoorganotrophic bacterium [9]. This species is characterized by the presence of C_16:1_ *ω*7*c* and C_18:1_ *ω*7*c* as major fatty acids, ubiquinone-7 (Q-7) and ubiquinone-8 (Q-8) as major respiratory quinones, and phosphatidylethanolamine, diphosphatidylglycerol, phosphatidylglycerol, and aminophospholipid as major polar lipids [9]. In the present study, we report the isolation of a bacterium, the designated strain IMCC34845^T^, from a freshwater stream. Based on the phylogenetic, genomic, physiological, and chemotaxonomic characteristics of the strain, we propose the inclusion of strain IMCC34845^T^ in the genus *Zwartia* as a novel species. The taxonomic evidence collected from this study also confirms that ‘Achromobacter panacis’ KCTC 42751^T^ [13] is clearly distinct from the genus *Achromobacter* and should, therefore, be transferred to the genus *Zwartia*.

## 2. Materials and Methods

### 2.1. Isolation and Maintenance

Strain IMCC34845^T^ was isolated from a freshwater sample collected at Wangsuk stream, a tributary of the Han River, Republic of Korea. The sampling station is situated downstream of a wetland area, which serves as both the site for effluent discharge from sewage treatment and retention of water within the wetland for 24 h prior to its introduction into the stream. This attribute emphasizes the ecological value of the site, thereby reinforcing its essential role as a central urban habitat characterized by an elevated standard of water quality. A water sample (37°36′41.4″ N, 127°8′52.4″ E) was collected in June 2018, and an aliquot of the sample was inoculated onto Reasoner’s 2A (R2A) agar (BD Diagnostics, Le Pont de Claix, France). After incubation at 20 °C for 2 weeks, strain IMCC34845^T^ was isolated as a single colony, routinely cultured on R2A at 30 °C, and stored at −80 °C in 10% (*v*/*v*) glycerol suspension. For phenotypic and genomic comparisons, two closely related species, ‘Achromobacter panacis’ KCTC 42751^T^ and *Z. hollandica* LF4-65^T^, were obtained from the Korean Collection for Type Cultures (KCTC) and the original isolator, respectively. These species were used as experimental controls for further characterization.

### 2.2. 16S rRNA Gene Sequence Analysis

The 16S rRNA gene of strain IMCC34845^T^ was amplified via PCR using the universal bacterial primers 27F and 1492R [14] and sequenced using the Sanger sequencing method (Biofact Co., Daejeon, Korea). The resultant 16S rRNA gene sequence of strain IMCC34845^T^ (1448 bp) was identified using BLASTn searches in the GenBank database and the 16S-based ID in EzBioCloud [15]. For phylogenetic analysis, 16S rRNA gene sequences of strain IMCC34845^T^ and closely related type strains were aligned using the Silva Incremental Aligner and imported into the ARB software v.6.06 [16]. Based on the aligned sequences of strain IMCC34845^T^ and the phylogenetically related species, phylogenetic trees were generated using the following tree-inferring methods implemented in the MEGA X program [17]: the maximum likelihood method [18] with the Tamura–Nei model, the neighbor-joining method [19] with Jukes–Cantor correction, and the minimum evolution method [20] with Jukes–Cantor correction. The topology of the phylogenetic trees was evaluated by using bootstrap analyses based on 1000 random replicates [21].

### 2.3. Genome Analysis

Genomic DNA was extracted from strains IMCC34845^T^ and KCTC 42751^T^ using the DNeasy Blood & Tissue kit (Qiagen,) following the manufacturer’s instructions. Whole-genome sequencing of strain IMCC34845^T^ was conducted on the Illumina HiSeq platform (2 × 150 paired-end run) by Macrogen Co. (Seoul, Korea), while strain KCTC 42751^T^ was sequenced on the Illumina NovaSeq platform (2 × 150 paired-end run) by DNALink Co. (Seoul, Korea). The raw sequencing reads were trimmed using the BBMap tool (https://sourceforge.net/projects/bbmap/, accessed on 12 May 2023) and assembled using SPAdes version 3.15.5 [22]. The quality of the assembled genome was evaluated using CheckM [23].

To determine genomic relatedness and perform comparative genomic analysis, the genome sequences of *Z. hollandica* LF4-65^T^ (GenBank accession, JAHXRI000000000), *S. amnicola* NBD-18^T^ (JAAGRN000000000), and *J. montanilacus* MWH-P2sevCIIIb^T^ (PVTV00000000) were downloaded from GenBank. The genome relatedness value was determined using the average nucleotide identity (ANI) and digital DNA-DNA hybridization (dDDH) values through the OrthoANI algorithm [24] and genome-to-genome distance calculator (GGDC 2.1) [25], respectively. To infer a genome-based phylogenetic tree, 81 universal bacterial core genes were extracted using the up-to-date bacterial core gene set and pipeline (UBCG2) [26] and employed to reconstruct the phylogenetic tree using RAxML [27]. Pan- and core-genome analyses were performed using the GET_HOMOLOGUES software v.3.3.2 [28] with the orthoMCL algorithm, utilizing protein sequences predicted by Prokka [29]. Prior to analyzing the distribution of Clusters of Orthologous Groups (COGs) categories and metabolic pathways, protein-coding genes of each genome were obtained using Prokka [29]. Assignment of the proteins into COG functional categories [30] was performed using RPS-blast search (e-value cutoff, 0.01) [31] against the COG profile database. Metabolic pathways were predicted using BlastKOALA [32], and the results were confirmed with KofamKOALA [33].

### 2.4. Physiological and Chemotaxonomic Analyses

Phenotypic characteristics of strain IMCC34845^T^ were determined following routine cultivation on R2A for 5 days at 30 °C. Cellular morphology was examined using transmission electron microscopy (TEM, CM200; Philips, Tokyo, Japan) with cells stained with 2% (*w*/*v*) uranyl acetate on a carbon-coated copper grid. Gliding motility was tested by observing bacterial spread through semi-solid agar (R2A with 0.3% agar). The Gram test was performed using the Rye non-staining KOH technique [34]. Catalase activity was tested with 3% (*v*/*v*) hydrogen peroxide, and oxidase activity was examined using Kovac’s reagent (bioMérieux, Marcy-l’Étoile, France). Cellular growth was assessed on various bacteriological media, including R2A agar, nutrient agar (NA), marine agar (MA), tryptic soy agar (TSA), and plate count agar (PCA). Growth at different temperatures (4, 10, 15, 20, 25, 30, 35, and 40 °C) and pH ranges (pH 4.0–10.0, at intervals of 1.0) was investigated in R2A broth. The pH was adjusted using the following buffering system: citrate buffer (for pH 4.0), MES buffer (for pH 5.0), MOPS buffer (for pH 6.0), HEPES buffer (for pH 7.0), and CHES buffer (for pH 8.0–11.0). Salt tolerance was monitored in R2A broth supplemented with 0–3% (*w*/*v*) NaCl at intervals of 0.5%. Cellular growth was measured via optical cell density at 600 nm using a UV-Vis spectrophotometer (UV2600; Shimazu). Growth under anaerobic condition was monitored using the GasPak™ EZ Anaerobe Pouch System with Indicator (BD Diagnostics) for up to 4 weeks. Degradation of macromolecules was tested on R2A agar supplemented with macromolecules, including casein (3% skimmed milk, *w*/*v*), CM-cellulose (1%, *w*/*v*), colloidal chitin (1%, *w*/*v*), starch (1%, *w*/*v*), Tween 20 (1%, *v*/*v*), and Tween 80 (1%, *v*/*v*), and confirmed by the formation of distinct zones around the colonies after adding appropriate solutions or observing directly [35]. Degradation of DNA was tested using DNase test agar (BD Diagnostics,). H_2_S production was tested using triple sugar iron agar (BD Diagnostics), with confirmation by the emergence of black precipitates. For other biochemical tests, API 20NE, API ZYM strips (bioMérieux), and GEN Ⅲ MicroPlate (Biolog, Fremont, CA, USA) were used following the manufacturer’s instructions.

For the analysis of cellular fatty acid methyl esters (FAME), cells were harvested from colonies grown on the third quadrant sectors of the plates after incubation on R2A at 30 °C for 5 days. The FAME profile was analyzed using gas chromatography (Agilent 7890 GC, Santa Clara, CA, USA) with the Sherlock Microbial Identification System version 6.1 (MIDI) and a TSBA6 database [36]. Respiratory isoprenoid quinone was extracted following the procedures described by Minnikin et al. [37] and examined via reverse-phase partition chromatography [38] on Merck HPTLC RP-18F254 (Sigma-Aldrich, St. Louis, MO, USA) reverse-phase thin-layer plates. For the extraction and separation of polar lipids, previously described methods [37] were employed, and two-dimensional thin-layer chromatography (TLC) was performed on silica gel 60 F254 plates (Merck, Rahway, NJ, USA). Visualization of all polar lipids on the TLC plates was achieved by spraying with 10% (*w*/*v*) molybdatophosphoric acid (Sigma-Aldrich). Specific lipids containing functional groups were identified by spraying 0.2% (*w*/*v*) ninhydrin (Sigma-Aldrich) for aminolipids, 1.3% (*w*/*v*) molybdenum blue (Sigma-Aldrich) for phospholipids, 2.4% (*w*/*v*) alpha-naphthol solution for glycolipids, and Dragendorff solution for choline.

## 3. Results

### 3.1. 16S rRNA Gene Phylogeny

**Figure 1:**
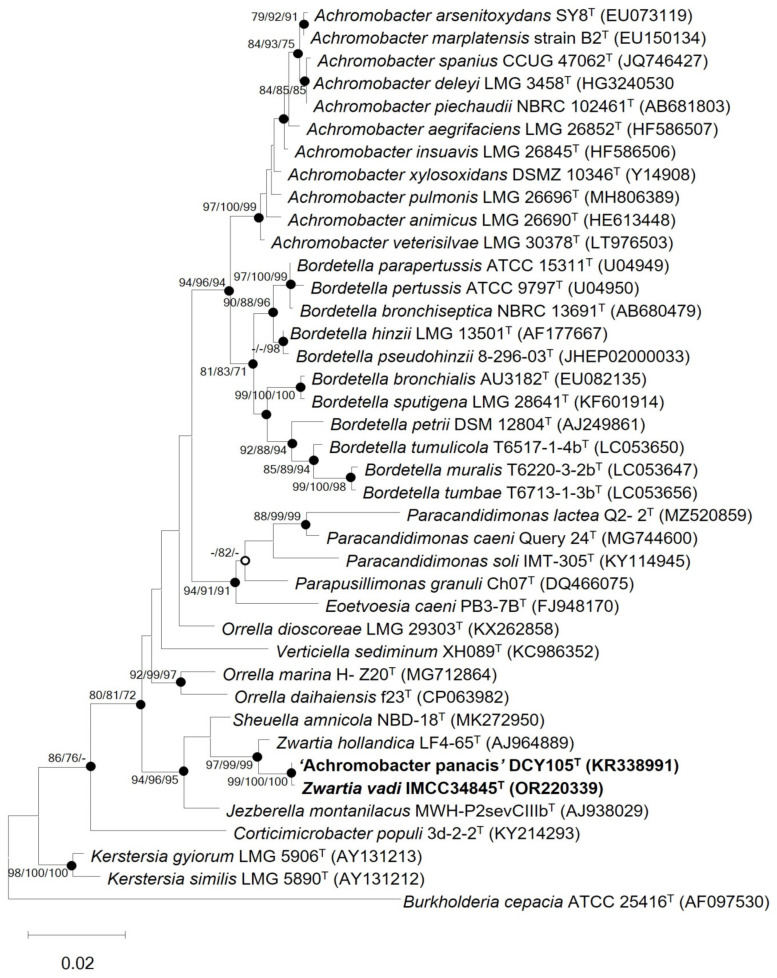
Maximum likelihood phylogenetic tree based on 16S rRNA gene sequences showing the relationships among strain IMCC34845^T^, related type strains of the GKS98 cluster, and other species of the family *Alcaligenaceae*. Bootstrap values (expressed as percentages of 1000 replications) over 70% are shown at nodes for maximum likelihood, neighbor-joining, and minimum evolution methods, respectively. Filled circles indicate that the corresponding nodes were recovered by all treeing methods. Open circles indicate that the corresponding nodes were recovered by any two out of three methods. *Burkholderia cepacia* ATCC 25416^T^ (AF097530) was used as an outgroup. Bar, 0.02 substitutions per nucleotide position.

The 16S rRNA gene sequence similarity analyses showed that strain IMCC34845^T^ had the closest similarity to ‘A. panacis’ DCY105^T^ (100%), followed by *Z. hollandica* LF4-65^T^ (98.9%) and *S. amnicola* NBD-18^T^ (98.6%). In all the 16S rRNA gene-based phylogenetic trees generated using three tree-inferring methods, strain IMCC34845^T^ formed a robust clade with ‘A. panacis’ DCY42751^T^ and *Z. hollandica* LF4-65^T^ (Figure 1, Figures S1 and S2), which were supported by high bootstrap values, indicating that they belong to the genus *Zwartia*.

### 3.2. Whole Genome Analysis and Genomic Relatedness

The draft genome sequence of strain IMCC34845^T^ was 3,232,556 bp long with 443× genome coverage, comprising 72 contigs with 51.5% of DNA G+C (Table 1). The genome completeness and contamination values estimated by CheckM were 100% and 0.12%, respectively. The annotated genome contained a single copy of the 16S rRNA gene, which exhibited 100% similarity to the amplified 16S rRNA gene sequence. The general genomic features of IMCC34845^T^, KCTC 42751^T^, and LF4-65^T^, annotated using Prokka, are summarized in Table 1.

The OrthoANI and dDDH values between strains IMCC34845^T^ and KCTC 42751^T^ were 77.7% and 20.7%, respectively. Analyses based solely on 16S rRNA gene sequences fall short in effectively distinguishing bacterial species exhibiting substantial sequence resemblance. Consequently, ANI values of 95–96% have gained wide recognition as the threshold equivalent to a DDH value of 70% for species demarcation [39,40,41]. Remarkably, despite there being an evident 100% similarity in the 16S rRNA gene sequence between the two strains, these values remain below the recommended ANI and dDDH thresholds, unambiguously indicating that the two strains represent two distinct bacterial species. Similarly, even with a >98.7% 16S rRNA gene sequence similarity between the two strains and *Z. hollandica* LF4-65T, the genomic relatedness values ranged from 72.2% to 72.5% (for ANI) and from 18.3% to 18.5% (for dDDH), confirming that the two strains represent novel species apart from *Z. hollandica*. The low ANI values, despite the very high 16S rRNA gene sequence similarity within the genus Zwartia, highlights the significance of genome-based comparison for precise bacterial species classification. This aspect bears particular importance as microbiologists adhering to the 98.7% similarity criterion might dismiss new strains due to an excessive similarity with the described species, thereby potentially overlooking emergent species. The ANI and dDDH values among strain IMCC34845^T^ and the phylogenetically related species are summarized in Table 2.

**Figure 2:**
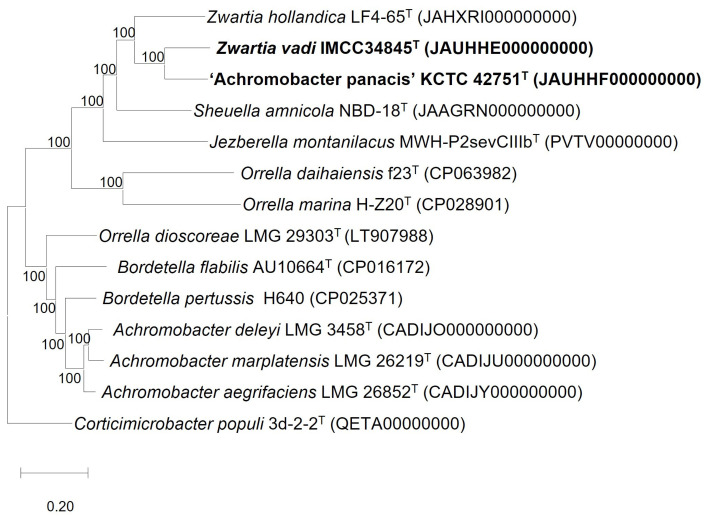
Phylogenomic tree based on concatenated multiple alignment of 81 genes showing the relationship between IMCC34845^T^ and closely related species of the family Alcaligeneceae. The tree was generated using UBCG2 pipeline with the concatenation of 81 gene sequences. GenBank accession numbers are shown in parentheses. Percentage bootstrap values are given at branching points. Bar, 0.2 substitutions per nucleotide position.

In the phylogenomic tree, strains IMCC34845^T^ and KCTC 42751^T^ formed a robust clade with *Z. hollandica* LF4-65^T^ and was distinct from other genera belonging to the GKS98 cluster, such as *J. montanilacus* MWH-P2sevCIIIb^T^ and *S. amnicola* NBD-18^T^ (Figure 2). These genomic DNA relatedness values and phylogenomic positions clearly indicated that strains IMCC34845^T^ and KCTC 42751^T^ represent each novel species of the genus *Zwartia*.

### 3.3. Comparative Genomic Analyses

**Figure 3:**
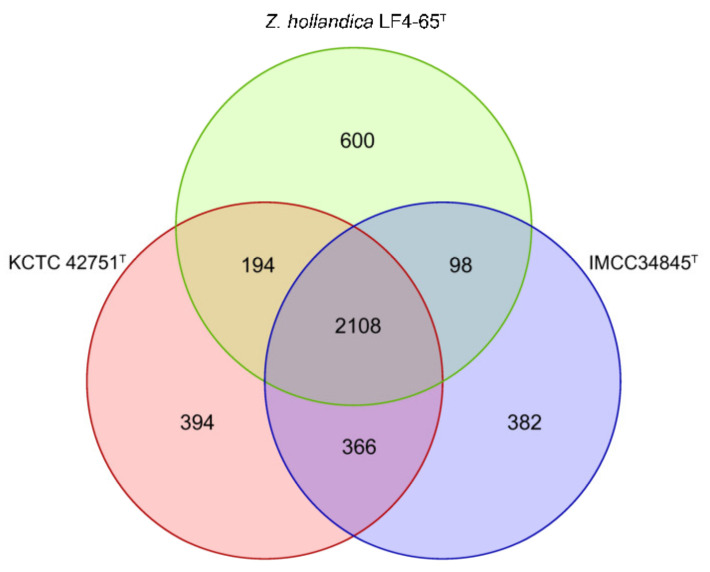
Venn diagram illustrating the number of unique and shared protein-coding genes in the genomes of three *Zwartia* species.

In the core- and pan-genome analyses, strains IMCC34845^T^, KCTC 42751^T^, and LF4-65^T^ shared 2108 protein clusters, accounting for only 68.8–71.4% of the protein-coding genes predicted in each genome (Figure 3). The genomes of the three strains encoded various central carbohydrate pathways, including the Embden–Meyerhof–Parnas pathway, gluconeogenesis, the tricarboxylic acid (TCA) cycle, the non-oxidative pentose phosphate pathway, and 5-phospho-α-d-ribose-1-diphosphate biosynthesis (PRPP biosynthesis). The three genomes also featured the SOX complex and the nitrogen regulation two-component system (NtrB/NtrC family). Additionally, the three strains carried the genes for carbonic anhydrase, which facilitates the hydration of dissolved CO_2_ into carbonic acid and dissociates to produce hydrogen carbonate at neutral pH levels. This suggests that the genus *Zwartia* may play a crucial role in sulfur, nitrogen, and carbon cycling in their habitats. However, there were differences in some nitrogen- and sulfur-related metabolic genes among the three genomes. Specifically, these differences included the presence of genes for nitrate reductase (NarGHI), glutamate dehydrogenase, cyanate lyase, sulfite reductase, and dimethyl-sulfone monooxygenase, which were encoded in the genomes of strains IMCC34845^T^ and KCTC 42751^T^ but were absent in *Z. hollandica* LF4-65^T^ (Table 1). The identification of orthologous genes based on the COG functional categories showed that translation, ribosomal structure, and biogenesis (J); cell wall/membrane/envelope biogenesis (M); energy production and conversion (C); amino acid transport and metabolism (E); coenzyme transport and metabolism (H); lipid transport and metabolism (I); and inorganic ion transport and metabolism (P) are enriched (≥5.0%) in the genome of IMCC34845^T^ (Table S1). 

### 3.4. Physiological and Chemotaxonomic Characteristics

The cells of strain IMCC34845^T^ were observed to be Gram-negative, aerobic, and motile by gliding. The TEM images revealed that the cells of strain IMCC34845^T^ were short, rod-shaped, and 1.1–1.6 × 0.4–0.5 μm in size (Figure S3), which were larger than those of *Z. hollandica* LF4-65^T^. However, no flagella were observed from the TEM images of the strain. The physiological and biochemical characteristics of strain IMCC34845^T^ are presented in Table 3 and the species protologue. Based on the phenotypic comparison, strains IMCC34845^T^ and KCTC 42751^T^ exhibited similar characteristics in terms of their morphology, motile by gliding, growth range of temperature and salinity, catalase, and most enzyme activities. However, they showed differences in the growth range of the pH, oxidase activity, and esculin hydrolysis ability (Table 3). Furthermore, strain IMCC34845^T^ differed from the type strain of *Z. hollandica* in cell size, several enzyme activities, and the carbon source utilization pattern (Table 3).

The major fatty acids (>10%) of strain IMCC34845^T^ were identified as C_16:0_ (38.4%) and C_17:0_ cyclo (32.4%), which closely resembled those of the the most closely related strain KCTC 42751^T^, with C_16:0_ and C_17:0_ cyclo contents of 37.3% and 30.5%, respectively (Table 4). However, *Z. hollandica* LF4-65^T^ exhibited lower proportions of C_17:0_ cyclo. *Z. hollandica* LF4-65^T^ contained higher proportions of C_16:0_ (25.0%), C_15:0_ iso (14.9%), and summed feature 3 (23.1%, C_16:1_ *ω*6*c* and/or C16:1 *ω*7*c*) as the major fatty acids (Table 4). The predominant respiratory quinones detected in strain IMCC34845^T^ were Q-7 and Q-8. The polar lipids of strain IMCC34845^T^ comprised diphosphatidylglycerol, phosphatidylethanolamine, phosphatidylglycerol, three unidentified phospholipids, an unidentified aminolipid, and three unidentified lipids (Figure S4), which were similar to those of ‘A. panacis’ KCTC 42751^T^ [13] and *Z. hollandica* LF4-65^T^ [9].

## 4. Taxonomic Conclusions

Phylogenetic inference, genome analyses, and chemotaxonomic characteristics supported the assignment of strain IMCC34845^T^ to the genus *Zwartia*. However, due to the low level of DNA-DNA relatedness and several different phenotypic characteristics compared to its close phylogenetic neighbors, it is evident that strain IMCC34845^T^ represents a novel species of the genus *Zwartia*. Therefore, we propose the name *Zwartia vadi* sp. nov. for strain IMCC34845^T^. Additionally, ‘Achromobacter panacis’ KCTC 42751^T^, which is a species with an invalidly published name, exhibited significant differences from all recognized *Achromobacter* species based on both 16S rRNA and genome sequence comparisons. Thus, we propose its reclassification as a novel species within the genus *Zwartia*, and the name *Zwartia panacis* comb. nov. is proposed.

## 5. Protologue

**Description of *Zwartia vadi* sp. nov.**

*Zwartia vadi* (va’di. L. gen. n. *vadi*, of a shallow place in a river).

The cells are Gram-stain-negative, strictly aerobic, motile by gliding, and short rod shaped. The cells are 1.1–1.6 µm long and 0.4–0.5 µm wide. The colonies are opaque, beige colored, circular with entire margins, and non-adherent to agar surface grown on R2A agar for 5 days. The cells can grow on nutrient agar and R2A agar. Growth occurs aerobically on R2A at 15–35 °C (optimum 30 °C) and at pH 7–10 (optimum pH 8) without NaCl. The oxidase and catalase activities are negative. All the macromolecules tested (casein, chitin, starch, CM-cellulose, Tween 20, Tween 80, and DNA) are not hydrolyzed. H_2_S is not produced. In the API 20NE test, the results are positive for nitrate reduction and esculin hydrolysis, but negative for indole production, glucose fermentation, arginine dihydrolase, urease, gelatinase, and β-galactosidase (PNPG). In the API ZYM test, the results are positive for alkaline phosphatase, esterase (C4), esterase lipase (C8), leucine arylamidase, valine arylamidase, cystine arylamidase, acid phosphatase, and naphthol-AS-BI-phosphohydrolase, but negative for lipase (C14), trypsin, *α*-chymotrypsin, *α*-galactosidase, β-galactosidase, β-glucuronidase, α-glucosidase, β-glucosidase, N-acetyl-β-glucosaminidase, α-mannosidase, and α-fucosidase. In the carbon source oxidation test (GEN Ⅲ microplate; Biolog), the results are positive for D-fructose-6-phosphate, L-histidine, glucuronamide, D-saccharic acid, methyl pyruvate, L-lactic acid, α-keto glutaric acid, D-malic acid, L-malic acid, Bromo-succinic acid, γ-amino-butyric acid, and acetoacetic acid. The major fatty acids are C_16:0_ and C_17:0_ cyclo. The major polar lipids are diphosphatidylglycerol, phosphatidylglycerol, phosphatidylethanolamine, three unidentified phospholipids, an unidentified aminolipid, and three unidentified lipids. The respiratory quinones detected are Q-7 and Q-8. The type strain, IMCC34845^T^ (=KCTC 92920^T^ = NBRC 114902^T^), was isolated from a water sample from Wangsuk stream, a tributary of the Han River, Republic of Korea. The length of the draft whole-genome sequence of the type strain is 3.2 Mbp. The DNA G+C content of the type strain is 51.5%. The GenBank accession number of the 16S rRNA gene sequence and the draft whole-genome sequence of the type strain are OR220339 and JAUHHE000000000, respectively.

**Description of *Zwartia panacis* comb. nov.**

Basonym: ‘Achromobacter panacis’ Singh et al., 2017.

The characteristics of the species are as given by Singh and colleagues for the type strain of ‘Achromobacter panacis’. The type strain is DCY105^T^ (=KCTC 42751^T^ = CCTCCAB 2015193^T^). The length of the draft whole-genome sequence of the type strain is 3.3 Mbp. The DNA G+C content of the type strain is 52.0%. The GenBank accession number of the draft whole-genome sequence of the type strain is JAUHHF000000000.

## Figures and Tables

**Table 1:** Genome characteristics of strains IMCC34845^T^ and KCTC 42751^T^ and the type species of the genus *Zwartia*. Strain: 1. strain IMCC34845^T^; 2. ‘Achromobacter panacis’ KCTC 42751^T^; and 3. *Zwartia hollandica* LF4-65^T^. +, Positive; −, negative.

	1	2	3
GenBank Accession number	JAUHHE000000000	JAUHHF000000000	JAHXRI000000000
Genome size (bp)	3,232,556	3,341,787	3,221,261
Contigs	72	54	29
N50	168,410	303,737	498,434
L50	7	4	3
DNA G+C content (%)	51.5	52.0	52.0
Protein-coding genes	3000	3118	3051
tRNA genes	40	40	43
rRNA genes	3	4	6
Related functions and genes:			
Nitrate reductase (NarGHI)	+	+	−
Glutamate dehydrogenase	+	+	−
Cyanate lyase	+	+	−
Sulfite reductase	+	+	−
Dimethyl-sulfone monooxygenase	+	+	−

**Table 2:** Average nucleotide identity (ANI) and digital DNA–DNA hybridization (dDDH) values among strain IMCC34845^T^ and phylogenetically related species of the GKS98 cluster. Strain: 1. strain IMCC34845^T^; 2. ‘Achromobacter panacis’ KCTC 42751^T^.

Strains	ANI (%)		dDDH (%)	
	1	2	1	2
IMCC34845^T^		77.7		20.7
‘Achromobacter panacis’ KCTC 42751^T^	77.7		20.7	
*Zwartia hollandica* LF4-65^T^	72.2	72.5	18.3	18.5
*Sheuella amnicola* NBD-18^T^	71.5	71.5	19.0	18.8
*Jezberella montanilacus* MWH-P2sevCIIIb^T^	70.0	69.9	19.3	18.8

**Table 3:** Differential characteristics of strain IMCC34845^T^ and the most closely related species of the genus *Zwartia*. Strain: 1. strain IMCC34845^T^; 2. ‘Achromobacter panacis’ KCTC 42751^T^; and 3. *Zwartia hollandica* LF4-65^T^. All strains are positive for Gram-stain and gliding motility; esterase, esterase lipase, leucine arylamidase, valine arylamidase, cystine arylamidase, acid phosphatase, α-glucosidase, and naphthol-AS-BI-phosphohydrolase; and D-fructose6-PO_4_, glucuronamide, L-lactic acid, D-malic acid, L-malic acid, and bromo-succinic acid. All species contained Q-7 and Q-8 as the major respiratory quinones and diphosphatidylglycerol, phosphatidylglycerol, and phosphatidylethanolamine as major polar lipids. +, positive; −, negative.

Characteristics	1	2	3
Isolation source	Freshwater	Rhizosphere of ginseng	Freshwater
Cell size	1.1–1.6 × 0.4–0.5	1.5–1.8 × 0.5–0.6	0.6 × 0.4 *
Motility	+	+	+(−) *
Temperature range (°C)	15–35	15–35	10–35(5–32) *
Optimum temperature (°C)	30	30	30
pH range	7–10	7–9	7–10
Salt tolerance (%)	0	0	0(0–0.3) *
Oxidase	−	+	+(−) *
API 20NE			
Nitrate reduction	+	+	−
Esculin hydrolysis	+	−	−
API ZYM			
Alkaline phosphatase	+	+	−
GEN Ⅲ MicroPlate (Biolog)			
L-histidine, D-saccharic acid, methyl pyruvate, and *γ*-amino-butryric acid	+	+	−
Acetoacetic acid	+	−	−
D-fucose, L-fucose, L-galactonic acid lactone, β-hydroxy-D, and L-butyric acid	−	−	+

* Data are from Hahn et al. [9].

**Table 4:** Comparison of fatty acids of strain IMCC34845^T^ and closely related species of the genus *Zwartia*. Strain: 1. strain IMCC34845^T^; 2. ‘Achromobacter panacis’ KCTC 42751^T^; and 3. *Zwartia hollandica* LF4-65^T^. Data were obtained in this study. All strains were cultured for 5 days on R2A agar at 30 °C. −, not detected; Tr, traces (<1.0%). Major fatty acids (>10%) are shown in bold.

Fatty Acids	1	2	3
Saturated			
C_14:0_	2.1	1.7	2.0
C_16:0_	**38.4**	**37.3**	**25.0**
C_18:0_	2.1	1.3	4.2
Branched chain			
iso-C_13:0_	−	−	1.7
iso-C_14:0_	1.9	2.1	5.7
iso-C_15:0_	Tr	Tr	**14.9**
iso-C_15:0_ F	Tr	Tr	1.7
iso-C_16:0_	−	−	4.8
anteiso-C_15:0_	−	−	1.1
cyclo-C_17:0_	**32.4**	**30.5**	Tr
Hydroxy			
C_16:0_ 2OH	1.7	Tr	Tr
C_16:1_ 2OH	−	1.2	Tr
Summed feature *			
2	7.0	6.3	2.3
3	8.3	7.9	**23.1**
8	2.2	3.7	1.4
9	Tr	3.2	5.8

* Summed features are groups of several fatty acids that could not be separated using the MIDI system. Summed feature 2 comprises C_14:0_ 3-OH and/or iso-C_16:1_ I, summed feature 3 comprises C_16:1_ *ω*6*c* and/or C_16:1_ *ω*7*c*, summed feature 8 comprises C_18:1_ *ω*7*c* and/or C_18:1_ *ω*6*c*, and summed feature 9 comprises iso-C_17:1_ *ω*9*c* and/or C_16:0_ 10-methyl.

## Data Availability

The GenBank/EMBL/DDBJ accession number for the 16S rRNA gene sequence and the genome sequence of strain IMCC34845^T^ are OR220339 and JAUHHE000000000, respectively. The GenBank/EMBL/DDBJ accession number for the genome sequence of strain KCTC 42751^T^ is JAUHHF000000000.

## Supplementary Materials

The following supporting information can be downloaded at https://www.mdpi.com/article/10.3390/microorganisms11092150/s1, Figure S1: Neighbor-joining phylogenetic tree based on 16S rRNA gene sequences showing the relationships among strain IMCC34845^T^, related type strains of the GKS98 cluster, and other species of the family *Alcaligenaceae*; Figure S2: Minimum evolution phylogenetic tree based on 16S rRNA gene sequences showing the relationships among strain IMCC34845^T^, related type strains of the GKS98 cluster, and other species of the family *Alcaligenaceae*; Figure S3: Transmission electron micrographs of strains IMCC34845^T^ (A) and KCTC 42751^T^ (B) grown on R2A agar for 5 days at 30 °C; Figure S4: Two-dimensional thin-layer chromatogram of total polar lipids of strain IMCC34845^T^; Table S1: Clusters of Orthologous Groups (COGs) classification in the genomes of IMCC34845^T^, KCTC 42751^T^, and *Zwartia hollandica*.

## Abbreviations

R2A, Reasoner’s 2A; ANI, average nucleotide identity; dDDH, digital DNA-DNA hybridization; COGs, Clusters of Orthologous Groups; TEM, transmission electron microscopy; TLC, thin-layer chromatography; Q-7, ubiquinone-7; Q-8, ubiquinone-8; FAME, fatty acid methyl esters.
