# Supplementary material for: Zwartia vadi sp. nov., a Novel Species of the GKS98 Cluster Isolated from a Stream, and the Reclassification of ‘Achromobacter Panacis’ as Zwartia panacis comb. nov
Source: Microorganisms. 2023 Aug 24;11(9):2150. doi: 10.3390/microorganisms11092150 (PMC10535154; doi:10.3390/microorganisms11092150)
Supplement: Supplementary file 1 [file microorganisms-11-02150-s001.zip › microorganisms-2565524-supplementary.pdf]

Supplementary Figure S1. Neighbor-joining phylogenetic tree based on 16S rRNA gene sequences showing the relationships among strain IMCC34845^T, related type strains of the GKS98 cluster, and other species of the family *Alcaligenaceae*.

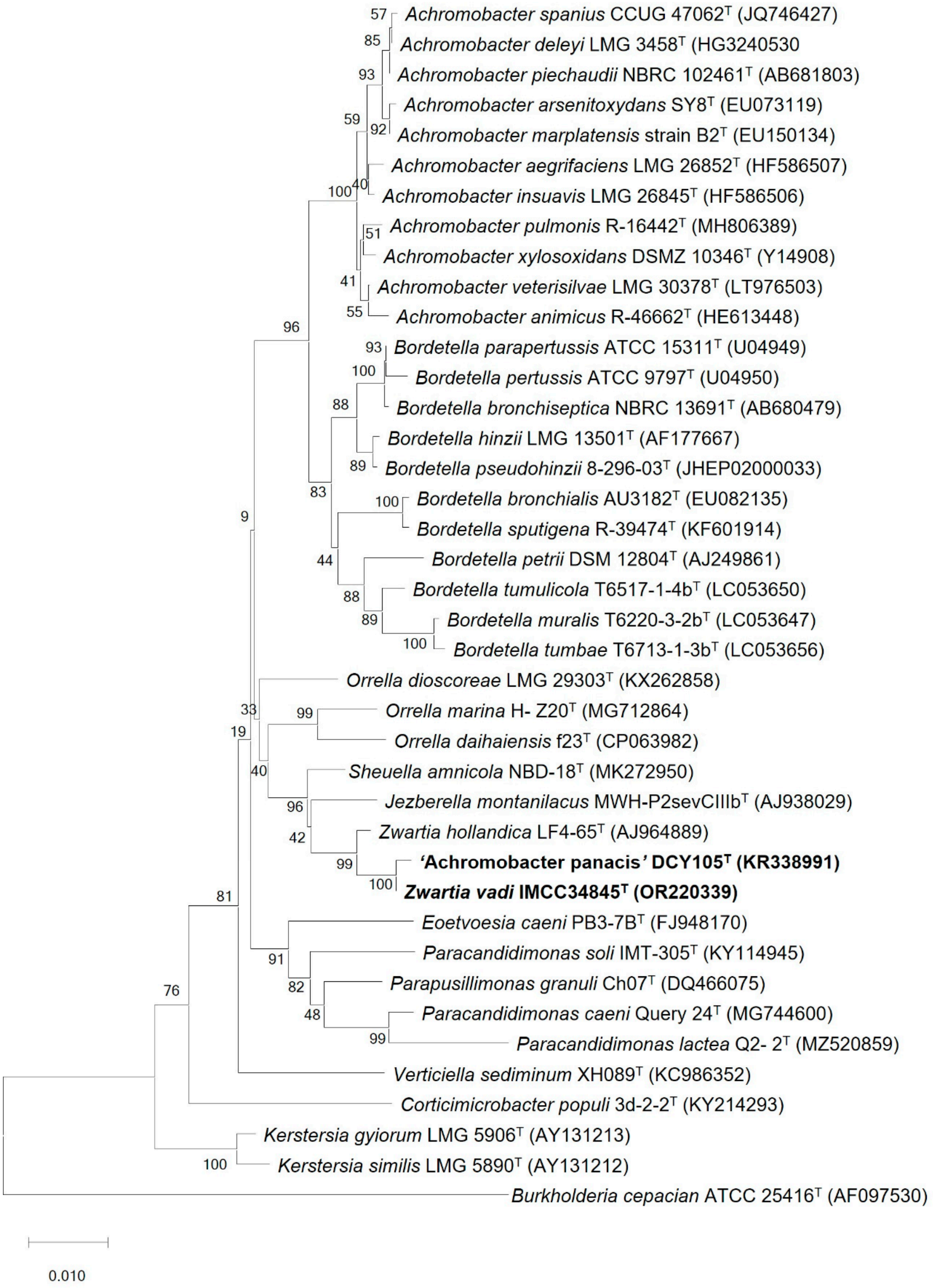

Supplementary Figure S2. Minimum-evolution phylogenetic tree based on 16S rRNA gene sequences showing the relationships among strain IMCC34845^{T}, related type strains of the GKS98 cluster, and other species of the family *Alcaligenaceae*.

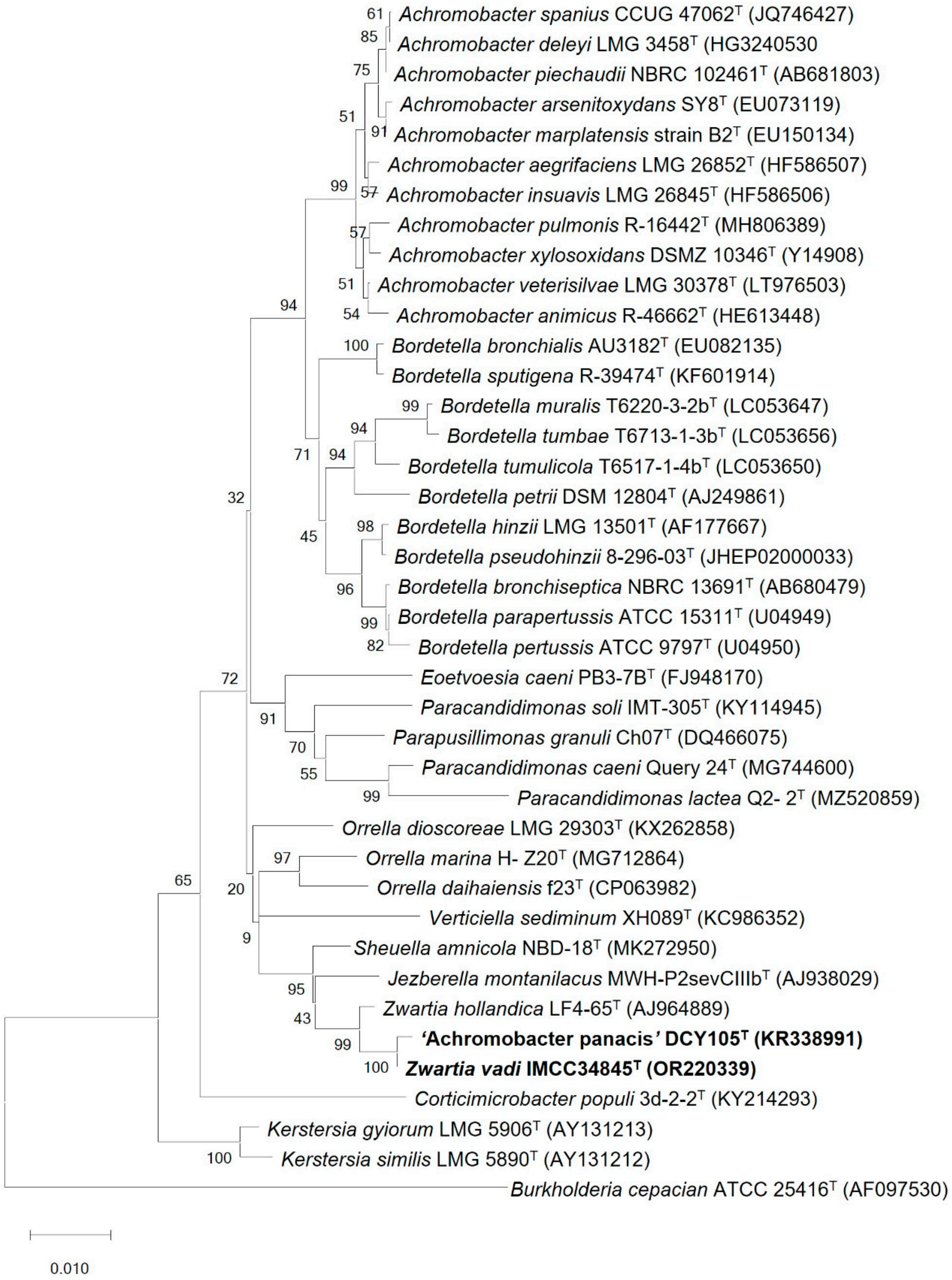

Supplementary Figure S3. Transmission electron micrographs of strains IMCC34845^{T} (A) and KCTC 42751^{T} (B) grown on R2A agar for 5 days at 30 ℃. Bar, 0.2 μm

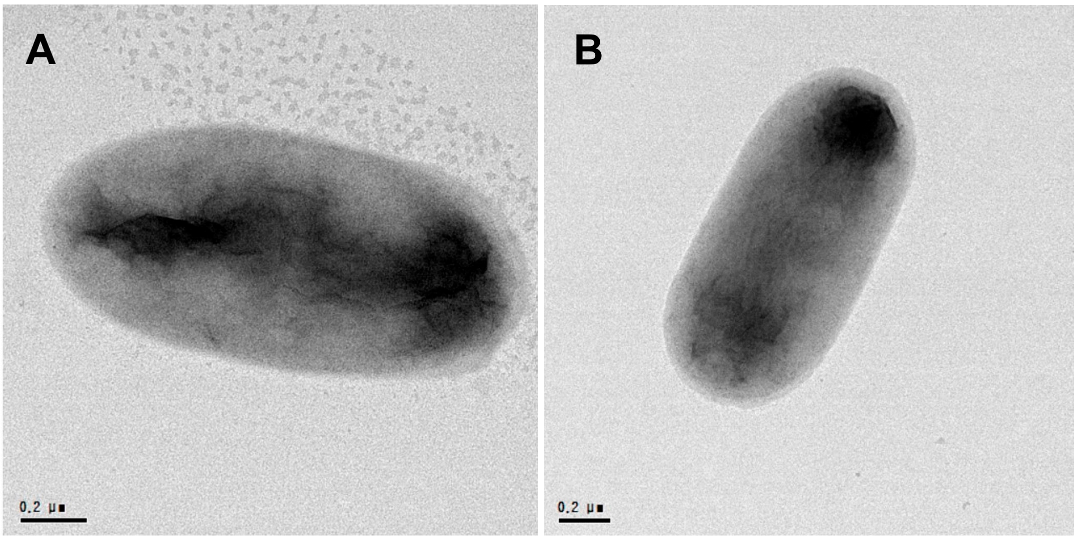

Supplementary Figure S4. Two-dimensional thin-layer chromatogram of total polar lipids of strain IMCC34845^{T}. Abbreviations: DPG, diphosphatidylglycerol; PG, phosphatidylglycerol; PE, phosphatidylethanolamine; PL1–3, unidentified phospholipids; AL, unidentified aminolipid; L1–3, unidentified lipids.

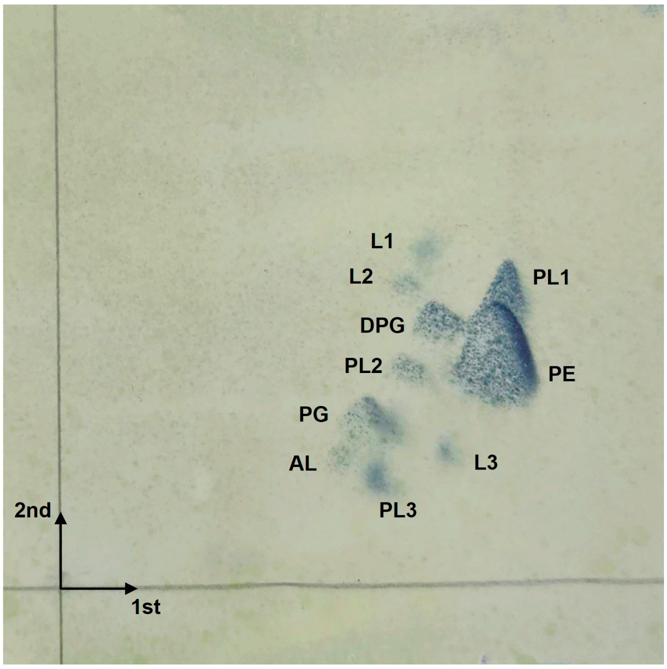

Supplementary Table S1. Cluster of Orthologous Groups (COG) classification in the genomes of IMCC34845^T, KCTC 42751^T, and type species of the genus *Zwartia*.

Strain: 1. strain IMCC34845^T; 2. 'Achromobacter panacis' KCTC 42751^T; 3. *Zwartia hollandica* LF4-65^T. Numbers indicate the percentage.

Code	Description	1	2	3
J	Translation, ribosomal structure, and biogenesis	7.7	7.2	8.0
K	Transcription	3.8	4.2	3.2
L	Replication, recombination, and repair	3.8	3.6	3.4
D	Cell cycle control, cell division, chromosome partitioning	1.5	1.7	2.2
V	Defense mechanisms	1.8	1.7	1.6
T	Signal transduction mechanisms	3.6	3.5	2.7
M	Cell wall/membrane/envelope biogenesis	7.5	7.6	7.7
U	Intracellular trafficking, secretion, and vesicular transport	1.4	1.0	1.1
O	Posttranslational modification, protein turnover, chaperones	4.4	4.5	4.0
C	Energy production and conversion	11.8	12.4	12.0
G	Carbohydrate transport and metabolism	5.7	5.5	7.3
E	Amino acid transport and metabolism	10.0	10.4	10.8
F	Nucleotide transport and metabolism	2.2	2.2	2.1
H	Coenzyme transport and metabolism	5.3	5.4	5.3
I	Lipid transport and metabolism	8.4	7.5	8.1
P	Inorganic ion transport and metabolism	5.3	5.7	5.1
Q	Secondary metabolites biosynthesis, transport, and catabolism	2.5	2.7	2.6
R	General function prediction only	7.2	7.5	7.3
S	Function unknown	4.4	4.3	4.4
